# Colon specific delivery of miR-155 inhibitor alleviates estrogen deficiency related phenotype via microbiota remodeling

**DOI:** 10.1080/10717544.2022.2108163

**Published:** 2022-08-07

**Authors:** Lianbi Zhao, Tian Zhou, Jianmei Chen, Wenbin Cai, Ruijing Shi, Yunyou Duan, Lijun Yuan, Changyang Xing

**Affiliations:** Department of Ultrasound Diagnostics, Tangdu Hospital, Fourth Military Medical University, Xi’an, China

**Keywords:** Microbiota, cardiac function, miRNAs, colon specific drug delivery, metabolic syndrome

## Abstract

Compelling data have indicated menopause-associated increase in cardiovascular disease in women, while the underlying mechanisms remain largely unknown. It is established that changes of intestinal microbiota affect cardiovascular function in the context of metabolic syndrome. We here aimed to explore the possible link between host intestinal function, microbiota, and cardiac function in the ovariectomy (OVX) mouse model. Mice were ovariectomized to induce estrogen-related metabolic syndrome and cardiovascular defect. Microbiota was analyzed by 16s rRNA sequencing. miRNA and mRNA candidates expression were tested by qPCR. Cardiac function was examined by echocardiography. Colon specific delivery of miRNA candidates was achieved by oral gavage of Eudragit S100 functionalized microspheres. In comparison with the sham-operated group, OVX mice showed compromised cardiac function, together with activated inflammation in the visceral adipose tissue and heart. *Lactobacillus* abundance was significantly decreased in the gut of OVX mice. Meanwhile, miR-155 was mostly upregulated in the intestinal epithelium and thus the feces over other candidates, which in turn decreased *Lactobacillus* abundance in the intestine when endocytosed. Oral delivery of miR-155 antagonist restored the protective microbiota and thus protected the cardiac function in the OVX mice. This study has established a possible regulatory axis of intestinal miRNAs-microbiota-estrogen deficiency related phenotype in the OVX model. Colon specific delivery of therapeutic miRNAs would possibly restore the microbiota toward protective phenotype in the context of metabolic syndrome.

## Introduction

Accumulating evidences underline the importance of the intestinal microbiota, which is considered as a kind of ‘external organ’, for the health of the host organism. It has been shown that the intestinal microbiota is a complex and dynamic ecosystem that influences host immunity and metabolism beyond the intestine (Kazemian et al., 2020; Liuzzo & Galiuto, 2021). The epidemic studies suggest that strong links between gut microbiota and cardiovascular disease (CVD) (Tang et al., 2017, 2019). For example, bacterial translocation and infiltration occur when the intestine is damaged (Chassaing et al., 2015). Besides, gut microbe-derived metabolites that are biologically active, such as trimethylamine N-oxide (TMAO), are now recognized as contributors to atherogenesis (Nam, 2019).

It has been well established that the risk for cardiovascular disease increases in women after menopause (Maas et al., 2021), which might be attributed to hormone change related obesity and the metabolic syndrome (MetS) (Barros & Gustafsson, 2011). Estrogen plays an important role in the control of energy homeostasis in females, while estrogen deficiency seems to have an unfavorable impact on mobilization of fatty acids, body fat distribution, and glucose-absorbing capacity of different tissues in post-menopaused women (Mauvais-Jarvis et al., 2013). Similarly, in ovariectomized mouse model, deprivation of estrogen leads to increases in fat mass, insulin resistance and cardiac dysfunction, further proving this point (Sharma et al., 2020; Hou et al., 2021). However, the detailed mechanism responsible for the metabolism change and thus cardiovascular disease in the context of estrogen deficiency, remains largely unknown. Estrogen plays important roles in the pathophysiological regulation of the intestinal functions. It is reported that estrogen receptors are expressed in enteric neurons, enteric glial cells, smooth muscle cells, and epithelial cells, indicating a role in regulating contractility of colon (D'Errico et al., 2018). Moreover, estrogen is found to be involved in the regulation of colonic epithelium function, including mucus secretion, gene expression, and exocytosis (Wada-Hiraike et al., 2006; Cuello-Carrion et al., 2010). On the other hand, the microbiota has recently been found to be shaped by the host intestine epithelium via fecal miRNAs (Liu et al., 2016). It has been revealed that fecal miRNA acts as a dominant player in the host control of the gut microbiota (Thomas, 2016). These miRNAs can enter bacteria, regulate bacterial gene transcripts, affect bacterial growth and thus shape the gut microbiota (Liu et al., 2016).

In view of the above literature, we speculate that estrogen deficiency would possibly change the secreted miRNAs in the intestine, which in turn shapes the microbiota in an unfavorable phenotype toward cardiovascular disease. In this study, the microbiota change, its effects on cardiac function and the possible underlying mechanisms in the processes were systemically analyzed in the ovariectomized (OVX) mouse model and postmenopausal women.

## Methods

Detailed methods are available in the online-only Supplemental Material. Requests by researchers to access the data, analytic methods, and study materials can be made to the corresponding author who manages the information.

### Mice husbandry

The female C57BL/6J mice from the experimental animal center of the Fourth Military Medical University (Xi’an, China) were housed and processed in accordance with the Institutional Animal Care and Use Committee in the Fourth Military Medical University. C57BL/6J mice were housed in plastic cages in a controlled environment animal facility at 22 ± 2 °C with a 12-hour light/dark cycle. Mice from the same litter were randomly divided into different groups, and different groups of mice were grown in different cages with each cage of less than 5 mice.

### OVX model

The OVX procedure was performed on 12-week-old C57BL/6J mice, and the age-matched C57BL/6J mice receiving a sham operation served as the controls. For the OVX surgery, the mice were anesthetized with an intraperitoneal injection of 1% sodium pentobarbital (40 mL/kg body weight). A 1-cm incision was made in the skin and back muscles parallel to the midline of the animal. Next, the ovaries were exposed, and the oviduct, including the ovarian blood vessels, was ligated followed by ovary removal. The incision in the back musculature was closed with nylon thread (Ethicon 4.0) and the skin was sutured, followed by disinfection. Throughout the surgical procedure, the mouse body temperature was maintained at 37°C with a heating pad. The same surgeon did all surgery procedures. All the mice with either OVX or sham operation were fed with normal chow diet for additional 8 weeks.

### Fecal microbiota transplantation and microsphere oral gavage

Fecal microbiota transplantation was done as described previously (Chassaing et al., 2015). Briefly, cecal contents from the normal control mice were suspended in 30% glycerol diluted in PBS (1.0 mL) and stocked at −80 °C until transplantation. Fecal microbiota transplantation (200 μL of fecal suspension) was orally delivered every other day in the mice after the sham or OVX operation (PBS as negative control), or treated with the negative control miRNA or miR-155 microspheres. The recipient mice didn’t receive any antibiotics treatment before or after transplantation. Transplanted mice at indicated time were subjected to cardiac function and inflammation analysis as previously described.

### Antibiotics treatment

To confirm whether the effects of colon specific delivery of miRNA antagonists rely on the colonic microbiome, miR-155 antagonists treated or control mice were additionally given penicillin plus vancomycin (1 mg antibiotic per g body weight per day) or no antibiotics (control) via drinking water. Eight weeks later, cardiac function and inflammation in both adipose tissue and heart were analyzed.

### Mouse echocardiography

Mouse echocardiography was done as previously described (Yuan et al., 2010). Briefly, the mice were anesthetized by using inhaled 1% to 2% isoflurane gas and 100% oxygen via a circuit anesthesia apparatus. The mouse’s core temperature was monitored using a rectal temperature probe, and an infrared heating lamp was used to maintain body temperature throughout the procedure. An electrocardiogram signal was also monitored through electrode pads on the heated platform. The heart rate was maintained at about 400 bpm by adjusting the isoflurane dose. The VisualSonics Vevo 2100 ultrasound instrument (VisualSonics Inc., Toronto, ON, Canada) armed with 30 MHz linear transducer was employed. The left ventricular (LV) systolic function was computed from the M-mode measurements. Pulsed Doppler studies of LV diastolic function were performed in the apical 4-chamber view with the Doppler cursor oriented parallel to the long-axis plane of the left ventricle. The sample volume was placed just below the level of the mitral annulus and adjusted to render the highest early diastolic flow velocity peak of the transmitral Doppler flow signal. The early and late diastolic peak velocity (E, A) and their ratio (E/A) were derived from the transmitral Doppler waveform.

### Bacterial growth and incubation with miRNAs

To analyze the effects of miR-155 on the growth of *Lactobacillus*, *Lactobacillus gasseri* (ATCC 33323) grown in a sterile MRS (de Man, Rogosa and Sharpe) medium at 37 °C for 12 h in the presence or absence of different doses of miR-155.

### qPCR analysis of the miRNA expression

RNA from tissues or bacteria was extracted using TRIzol, while RNA from the feces was isolated using Stool RNA kit (Omega, US). Reverse transcription reaction was performed on 2 μg of each sample with miRCute miRNA qPCR Detection kit (Tiangen, Beijing, China) according to the manual instruction. The reverse-transcribed cDNA was used as the template for the amplification of interested miRNAs. U6 serves as internal control for mammalian cells. The relative expression was calculated using the 2^-dd^Ct method. The primers were shown in Table S1.

### Uptake of miRNA by bacterium

To confirm the capacity of miRNAs entering the bacterium, *Escherichia coli* were grown aerobically in LB medium together with PBS or FITC-labeled miRNA for immunofluorescence analysis. In addition, miRNA uptake efficiency was also analyzed by qPCR analysis of the miRNA abundance. About 1 mL *E. coli* in aliquot were treated with negative control miRNA or miR-155 or let-7g, and 12 h later, the *E. coli* was harvested for lysis and miRNA transcription. An equal amount of the reverse-transcribed RNA was included for qPCR analysis. The Ct value of the miRNA was calculated and compared.

### Statistical analysis

The data were expressed as mean ± SEM otherwise indicated and the differences were analyzed by ANOVA with Tukey’s post hoc test or Student's *t*-test using Graphpad Prism 7.0. Significance was considered at *p* < .05.

## Results

### Decreased cardiac function and its relevance to the microbial change in OVX model

To systematically analyze the phenotype of menopause-related estrogen deficiency, female mice were subjected to sham or OVX operation (Figure S1A). Consistent with previous reports (Stubbins et al., 2012; Varghese et al., 2021), OVX mice had a greater body weight and fat mass percentage than the sham mice (Figure S1B, C), while the food intake had no significant differences between sham and OVX mice (Figure S1D). Moreover, brown adipose tissue (BAT), subcutaneous fat tissue (SAT), and omental adipose tissue (OAT) were all much larger in the OVX mice than that in the sham group (Figure S1E–G). HE staining revealed that net weight increase of BAT in OVX mice could be attributed to the whitening of BAT (Figure S1H). Consistent with the obese phenotype of OVX mice, expression of tumor necrosis factor α (*Tnfα*), macrophage inflammatory protein (*Mip1*), and monocyte chemoattractant protein-1 (*Mcp1*) was also significantly increased in the OAT in OVX mice (Figure S1I). Consistent with the increase of cardiovascular disease risks in menopaused women (Maas et al., 2021), the cardiac systolic and diastolic function was significantly compromised in the OVX group, as seen by the decreased short axis EF and E/A values (Figure S2A–C). Accordingly, there was increased expression of reactive oxygen species (ROS) (Figure S3A, B) and inflammatory genes, such as *Tnfα* and *Mcp1* (Figure S3F) in the heart. Flow cytometry analysis further revealed that more CD11b + leukocytes and M1 macrophage infiltration in the heart of OVX mice (Figure S3C–E).

**Figure 1.**
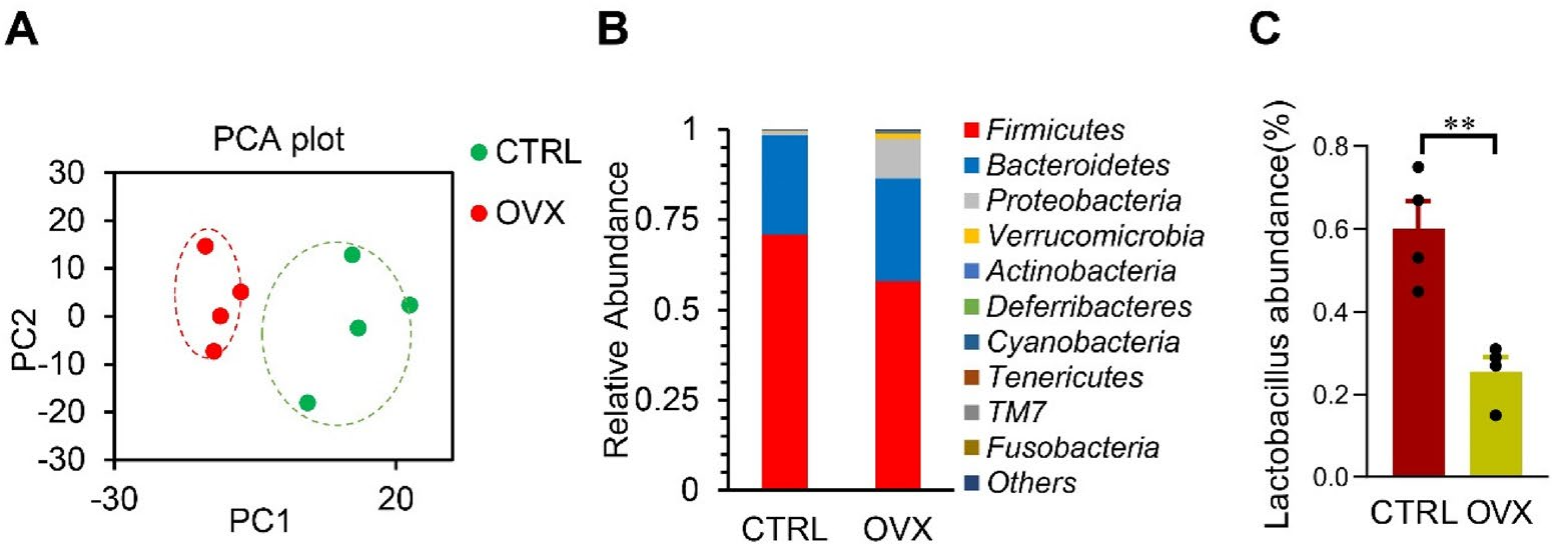
Changes of microbiota in OVX mice model. (A) Principal Component Analysis on the relative abundance of bacterial phylum. Each point represents a sample, plotted by the second principal component on the Y-axis and the first principal component on the X-axis, which was colored by group. (B) The relative abundance of OTUs (%) in the fecal bacterial community. (C) The relative abundance of *Lactobacillus* in the fecal bacterial community. Data are expressed as mean ± SEM. **p < .01.

**Figure 2.**
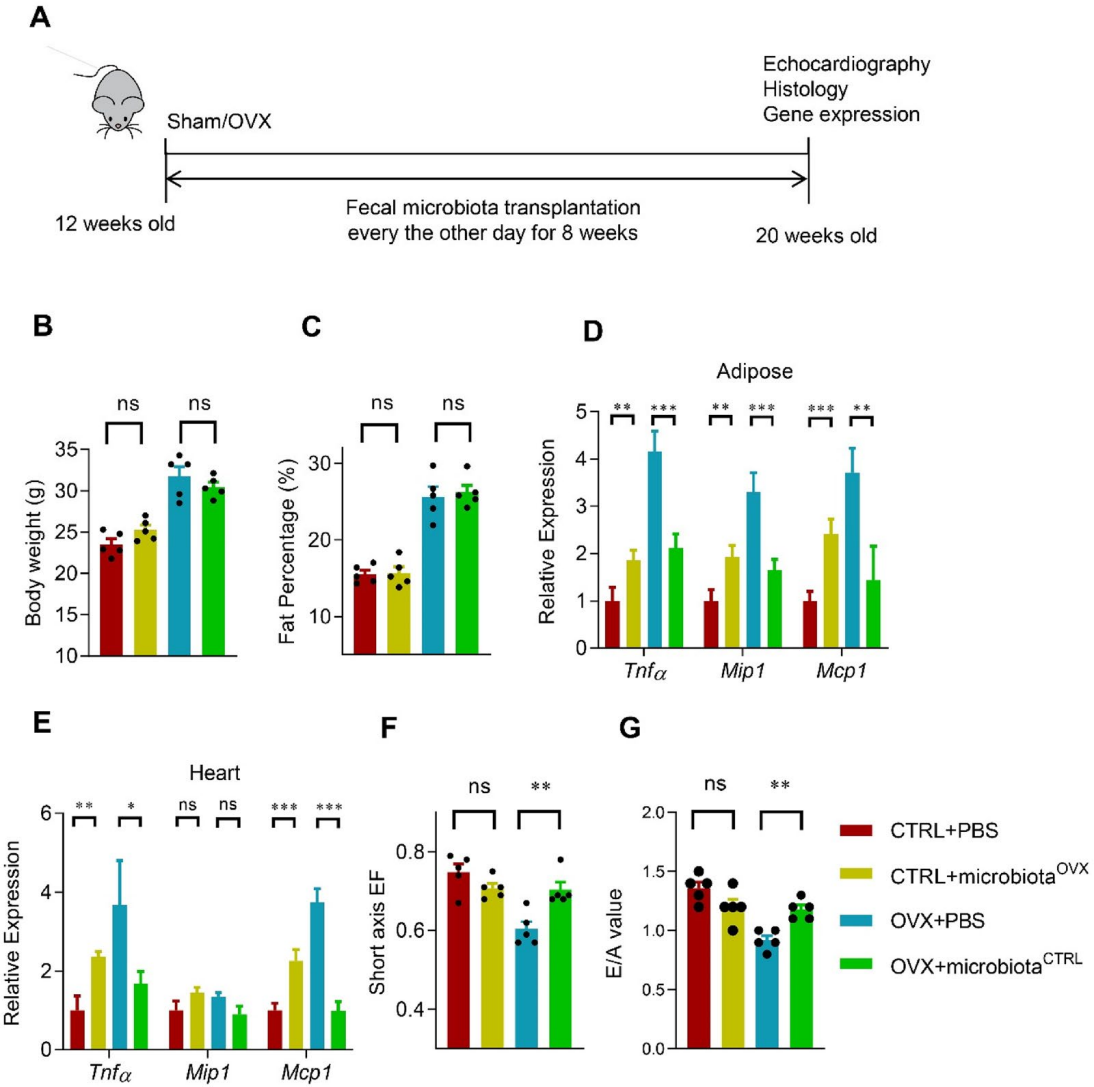
Transplantation of the normal fecal microbiota alleviates the inflammation and cardiac dysfunction in OVX mice. (A) Schematic representation of the experimental procedure. Fecal microbiota transplantation was done every other day after the sham or OVX operation. Body weight (B) and fat mass percentage (C) change after fecal transplantation. *n* = 5 for each group. (D, E) The expression of *Tnfα, Mip1,* and *Mcp1* in the visceral adipose tissue (D) and heart (E) in mice received indicated treatments, as detected by qPCR. qPCR was performed at least in triplicates. Data are expressed as mean ± SEM of three biological replicates. *, *p* < .05. Short axis EF (eject fraction) value (F) and E/A value (the ratio of early and late mitral diastolic peak velocity) (G) in mice received indicated treatments. Data are expressed as mean ± SEM of 5 mice per group. *p < .05, **p < .01, ***p < .001.

**Figure 3.**
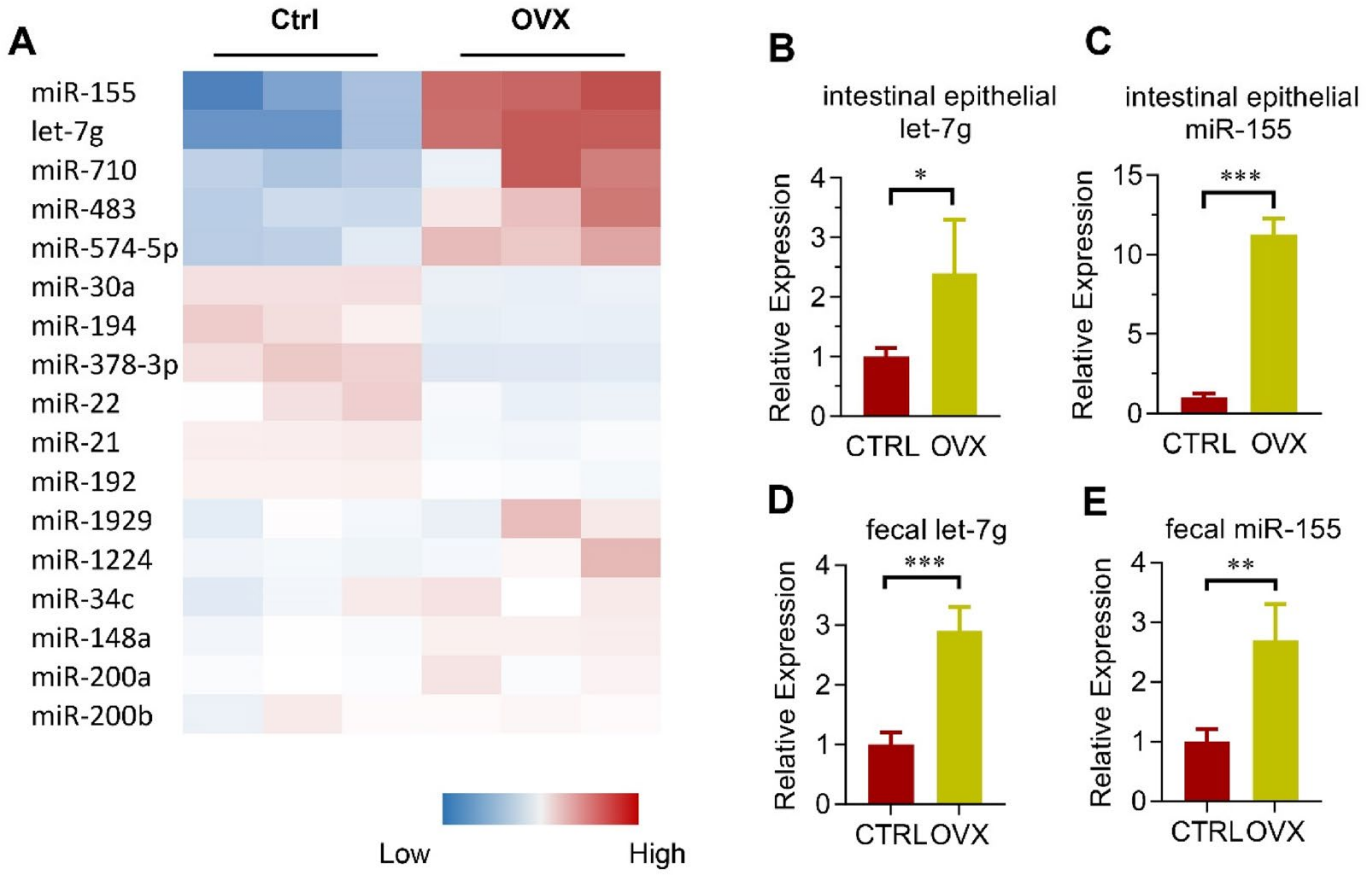
Involvement of miRNAs in microbiota remodeling in OVX mice. (A) Heatmap of the most abundant miRNAs expressed in the colon epithelium in control and OVX group. The color represents the normalized mean –dCt relative to internal control U6 in each mouse, with three mice in each group. (B, C) Validation of miR-155 (B) and let-7g (C) expression in the colon epithelium of control and OVX mice. Data are expressed as mean ± SEM of at least 3 biological replicates per group. (D, E) Relative expression of miR-155 (D) and let-7g (E) in the feces from the cecum of both control and OVX mice. Data are expressed as mean ± SEM of at least three biological replicates per group. *p < .05, **p < .01, ***p < .001.

Microbiota is found to be a novel modulator in obesity and inflammation (Belda et al., 2022; Dabke et al., 2019). To explore the role of microbiota in OVX mice-related phenotype change referred above, we thus analyzed the microbiota via 16s RNA sequencing. The microbiota was significantly altered in the OVX mice, as seen from the Principal Component Analysis (Figure 1A). Surprisingly, the relative abundance of the Bacteroidetes increased, whereas the Firmicutes decreased (Figure 1B). Notably, *Lactobacillus* abundance also decreased in the OVX group (Figure 1C).

To further verify the role of microbiota in the OVX related phonotype, we transplant the gut microbiota from the OVX mice to the control sham mice, and vice versa (Figure 2A). Fecal transplantation significantly changed the microbiota content in the recipient mice as expected. Fecal transplantation between the Sham and OVX mice slightly changed the body weight and fat mass percentage, although without significance (Figure 2B, C). However, transplantation of the OVX mice derived microbiota significantly increased the inflammatory genes in both the adipose tissue and hearts of the sham control mice, though the fold change was not as big as the OVX itself. In contrast, transplantation of the sham control mice derived microbiota significantly decreased the inflammatory genes in both the adipose tissue and heart of the OVX mice (Figure 2D, E). Consistent with the inflammation remodeling, normal fecal microbiota partially restored the cardiac function of OVX mice (Figure 2F, G). Unexpectedly, OVX fecal microbiota didn’t alter the cardiac function of control mice significantly (Figure 2F, G), suggesting that fat mass increase and microbiota remodeling might synergistically impair the cardiac function.

To further explore the role of decreased *Lactobacillus* abundance in the OVX-associated cardiac dysfunction, OVX mice were orally supplemented with *Lactobacillus* (Figure S4A). As expected, *Lactobacillus* supplementation significantly decreased the inflammatory genes in both the adipose tissue and heart of the OVX mice (Figure S4B, C), while the body weight and fat mass didn’t affect (Figure S4D, E). Consistent with the inflammation remodeling, *Lactobacillus* supplementation partially restored the cardiac function of OVX mice (Figure S4F, G), though the E/A value didn’t show a significant difference.

**Figure 4.**
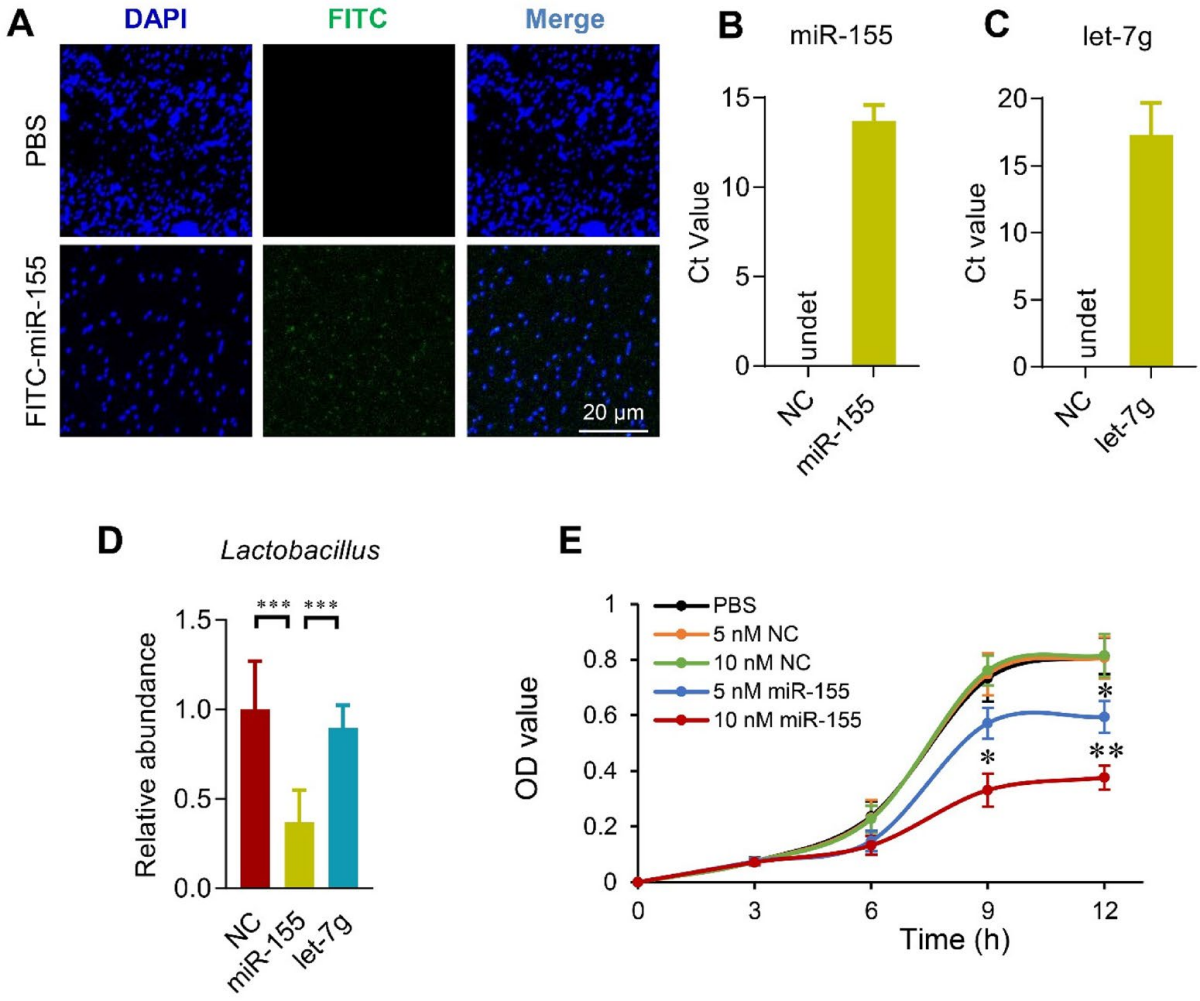
Host miRNAs enter the bacterium and regulate bacterial growth. (A) *E. coli* was cultured in the presence of FITC labeled miRNAs for 4 h, followed by fixation and DAPI staining. Images were acquired by confocal microscopy with a 100× objective. Data presented were representative of three independent experiments. Scale bar = 20 µm. Transfer of miR-155 (B) and let-7g (C) in the *E. coli* after 4 h incubation with control or indicated miRNAs. qPCR was performed at least in triplicates. NC is the miRNA mimic that can’t be detected by primers of miR-155 and let-7g. Data are expressed as mean ± SEM of three biological replicates. (D) *Lactobacillus gasseri* (ATCC 33323) growth in medium added with miR-155, let-7g or negative controls for 12 h. Data are expressed as mean ± SEM of three biological replicates. ***p < .001. (E) Growth curve of *Lactobacillus gasseri* (ATCC 33323) growth in medium added with negative control miRNA mimic or miR-155 at indicated doses. Data are expressed as mean ± SEM. *p < .05, 10 nM miR-155 vs. PBS, 5 nM NC, or 10 nM NC at 9 h; 5 nM miR-155 vs. PBS, 5 nM NC, or 10 nM NC at 12 h. **p < .01, 10 nM miR-155 vs. PBS, 5 nM NC, or 10 nM NC at 12 h.

All of these data raise the possibility that the microbiota might be remodeled toward an augmented inflammation signature in the context of OVX, and unraveling the factors remodeling the microbiota, especially the *Lactobacillus* abundance, would shed light on refining the microbiota toward better protective effects.

### Involvement of miRNAs in microbiota remodeling

In addition to defensins, microRNAs are also considered as one key factor of the host intestine that might change the microbiota (Salzman et al., 2010; Liu et al., 2016). With the easy manipulation and intervention of miRNAs, our preliminary interest was focused on the roles of miRNA candidates. The top 18 abundantly expressed miRNAs in the colon epithelium and feces (namely miR-155, let-7g, miR-1224, miR-30a, miR-34c, miR-483, miR-710, miR-1929, miR-194, miR-21, miR-22, miR-378-3p, miR-148a, miR-192, miR-200a, miR-200b, and miR-574-5p; based on the study by Liu et al. (2016)) were selected for further study. The miRNA expression was determined using qPCR analysis. Among the miRNAs, miR-155 and let-7g changed robustly in OVX mice (Figure 3A). Validation results further showed miR-155 and let-7g expression were up-regulated in OVX mice in both the intestinal epithelium (Figure 3B, C) and the feces (Figure 3D, E), when compared with that in the sham-operated mice.

### Increased miR-155 contributes to the decreased *Lactobacillus* in OVX model

To confirm the intestinal miRNAs do affect the microbiota, fluorescence labeled miRNAs were added in the bacteria culture medium and tracked by fluorescence microscope. Two hours after incubation with the *E. coli*, FITC labeled free miRNAs were found taken up by the bacteria, as shown from the robust green fluorescent signal inside the bacteria (Figure 4A). Furthermore, incubation of the *E. coli* with let-7g or miR-155 significantly increased the level of corresponding miRNAs in the *E. coli,* as detected by qPCR (Figure 4B, C). The capacity of the free miRNAs entering the bacteria further suggests that miRNAs might be a regulator of the microbiota and could be therapeutically targeted. In the following experiments, we explored whether let-7g or miR-155 could alter the abundance of *Lactobacillus*, as observed in the OVX mice. To this end, *Lactobacillus gasseri* (ATCC 33323) was grown in the presence of miR-155, let-7g or negative controls for 12 h. miR-155 greatly suppresses the growth of *Lactobacillus* (Figure 4D, E), while let-7g has no significant effects on the *Lactobacillus* growth (Figure 4D).

### Colon specific delivery of miR-155 antagonism protects the cardiac function via controlling the inflammation

**Figure 5.**
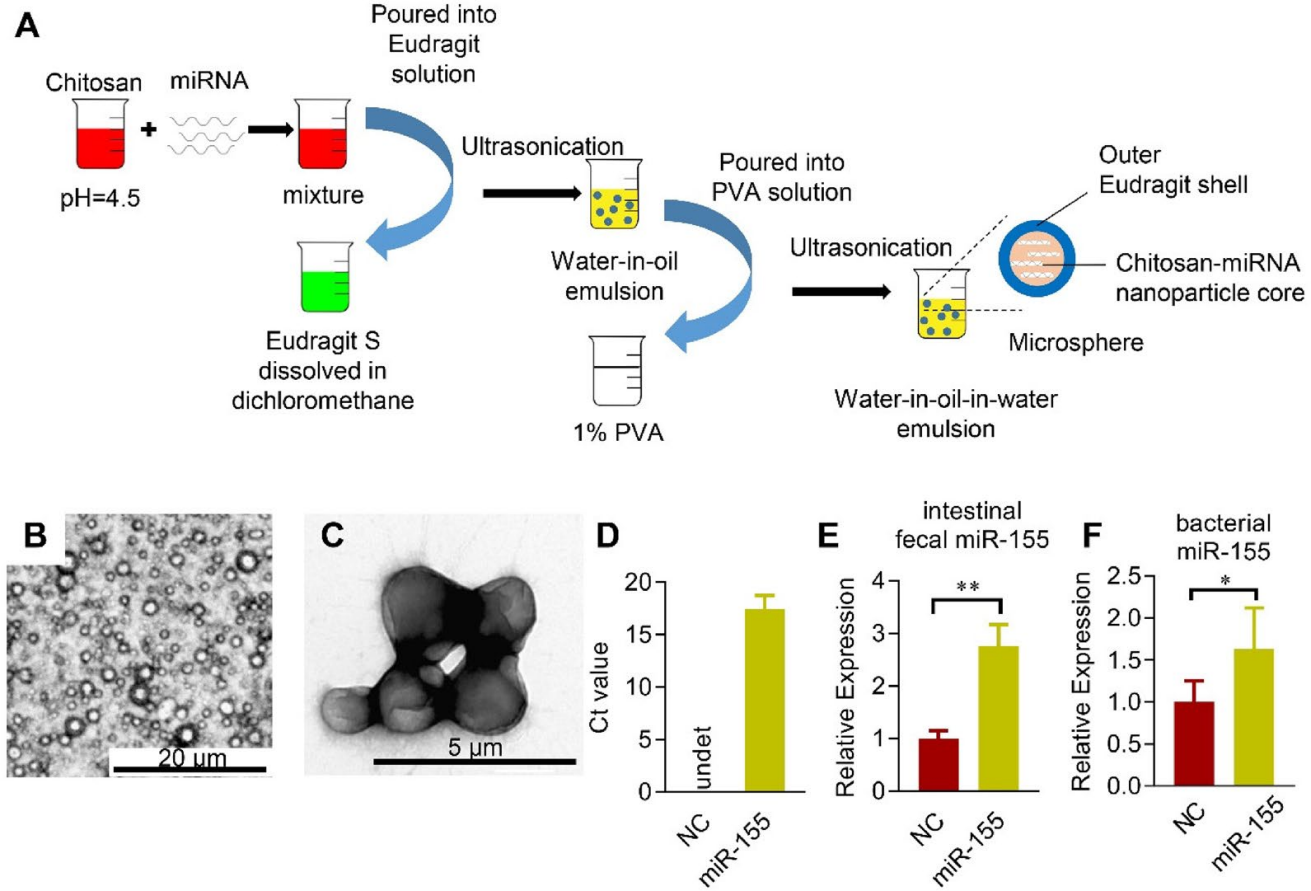
Microsphere mediated colon specific delivery of miRNAs. (A) Schematic illustration of the procedure how Eudragit S100 functionalized microsphere manipulated. miRNAs are incorporated in the Chitosan solution before mixed with the Eudragit S100: Methanol/dichloromethane solution, followed by ultrasonication. Next, the water-in-oil emulsion is poured into the PVA solution to form the water-in-oil-in-water (W1/O/W2) double emulsion. Representative bright-field microscope (B) and electron microscope (C) image of the fabricated Eudragit functionalized microspheres. (D) Encapsulation efficiency of miR-155 (D) loaded in the microsphere as assayed by qPCR. Enrichment of miR-155 in the feces (E) and cecal bacterium (F) from the cecum after orally delivered microspheres loaded with control or indicated miRNAs, NC is the miRNA mimic with no similar sequence as miR-155. qPCR was performed at least in triplicates. Data are expressed as mean ± SEM of three biological replicates. *p < .05, **p < .01.

To specifically alter the miR-155 levels in the colon, Eudragit S100 functionalized microsphere was employed, in which Eudragit S100 could protect the encapsulated cargos from the destruction by the acid in the stomach (Mehta et al., 2013). Eudragit S100 was coated on the chitosan-based microspheres (Figure 5A). The resultant microspheres were about 1–2 µm in diameter and evenly distributed (Figure 5B, C). qPCR analysis confirmed high encapsulation efficiency of miR-155 in the microsphere (Figure 5D). Moreover, gauge delivery of the Eudragit S100 functionalized microsphere resulted in miRNAs specifically released in the colon feces and bacterium (Figure 5E, F). For in vivo distribution analysis of the miRNA delivery specificity, cel-miR-54, which has no homology with miRNAs in human, mouse, and rat, was used. We encapsulated the cel-miR-54 into the microsphere and detected the transfer of cel-miR-54 into different organs in vivo via qPCR analysis. As expected, no obvious transfer of cel-miR-54 was found in the liver, spleen, adipose, heart, and other organs, except the intestinal epithelium (Figure S5). Taken together, we here developed a colon specific miRNA delivery strategy to alter the intestinal and fecal miRNA and to avoid degradation in the stomach and small intestine.

Next, we explored the effects of colon specific delivery of miR-155 on the microbiota, adipose and cardiac inflammation, and cardiac function (Figure S6A). miR-155 delivery significantly decreased the abundance of *Lactobacillus*, whereas had no significant effects on the total ratio of Bacteroidetes and Firmicutes (Figure S6B, C). In addition, body weight and fat mass percentage were not changed significantly (Figure S6D, E). Consistent with the decreased *Lactobacillus*, the expression of *Tnfα, Mip1,* and *Mcp1* in the adipose tissue and heart increased significantly, while *Lactobacillus* supplementation reduced the inflammatory gene induction (Figure S6F, G). Similar changes were seen in the immune cell infiltration (Figure S6H, I). Notably, the cardiac function was not obviously changed by miR-155 delivery alone (Figure S6J, K), suggesting that miR-155 might act synergistically with fat mass increase to impair cardiac function.

**Figure 6.**
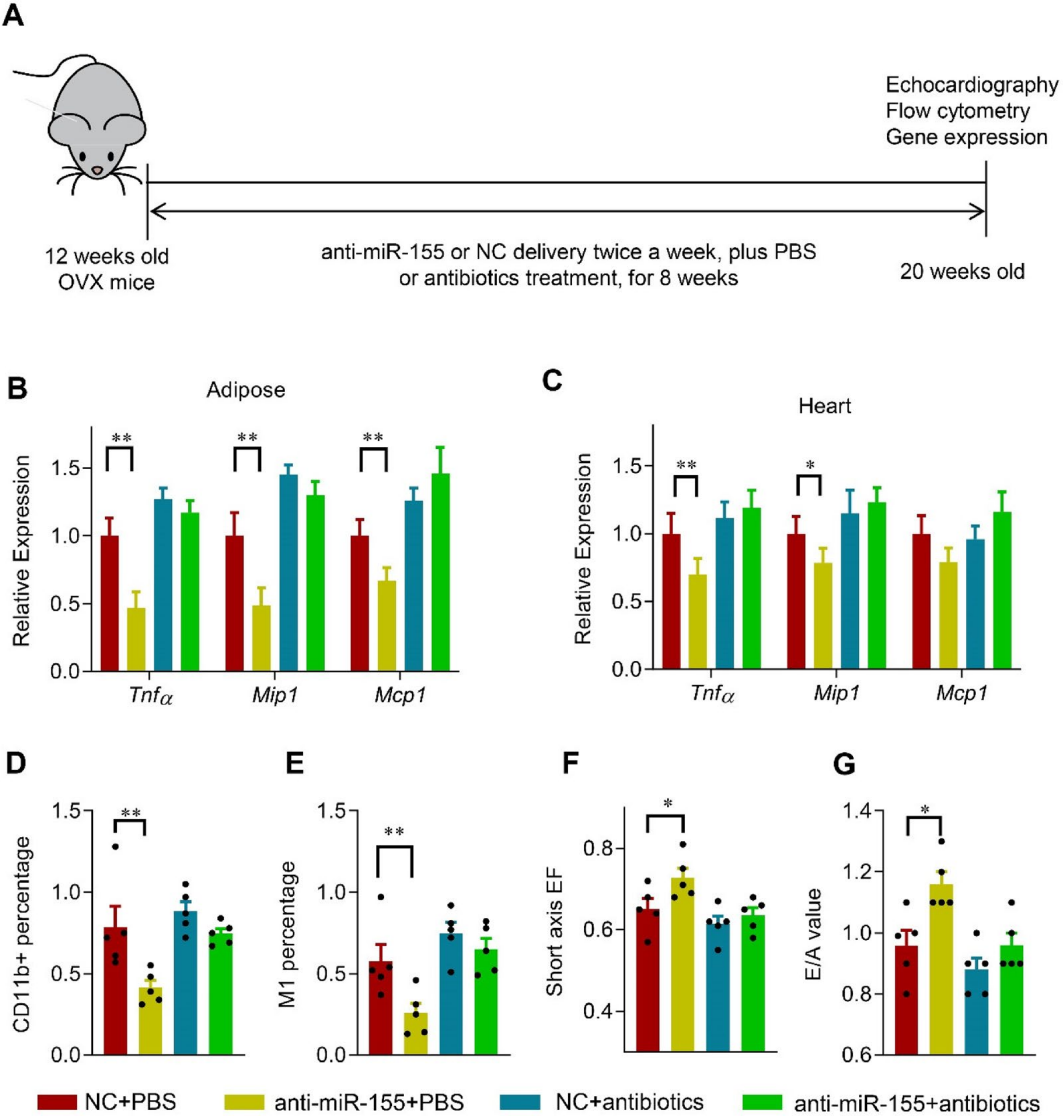
Oral delivery of miR-155 antagonism protects the heart in OVX mice via microbiota remodeling. (A) Schematic representation of the experimental procedure. OVX mice were treated with negative control or anti-miR-155 twice a week for 8 weeks, together with or without antibiotics in drinking water (1 mg antibiotic per g body weight per day). *n* = 5 for each group. (B, C) The expression of *Tnfα, Mip1,* and *Mcp1* in the adipose tissue (B) and heart (C) in mice received indicated treatments, as detected by qPCR. Data are expressed as mean ± SEM of three biological replicates. (D, E) CD11b + percentage (D) and M1 cell percentage (E) in mice treated as indicated. Data are expressed as mean ± SEM of 5 mice per group. (F, G) Short axis EF value (F) and E/A value (G) in mice received indicated treatments. Data are expressed as mean ± SEM of at least 5 mice per group. *p < .05, **p < .01.

In view of above data, we asked whether delivery of miR-155 antagonism would protect the heart from OVX-related damage via changing the microbiota (Figure 6A). Eudragit S100 functionalized microsphere mediated miR-155 antagonism delivery significantly increased the miR-155 antagonism in the colon feces and the bacteria (Figure S7A, B) in the OVX mice, although had no obvious change on the absolute level of miR-155 in both the feces and bacterium (Figure S7C, D). However, miR-155 related decrease of *Lactobacillus* in the OVX mice were nearly restored, with no obvious effects on either the ratio of Bacteroidetes/Firmicutes (Figure S7E, F), or the body weight and fat mass percentage (Figure S7G, H).

**Figure 7.**
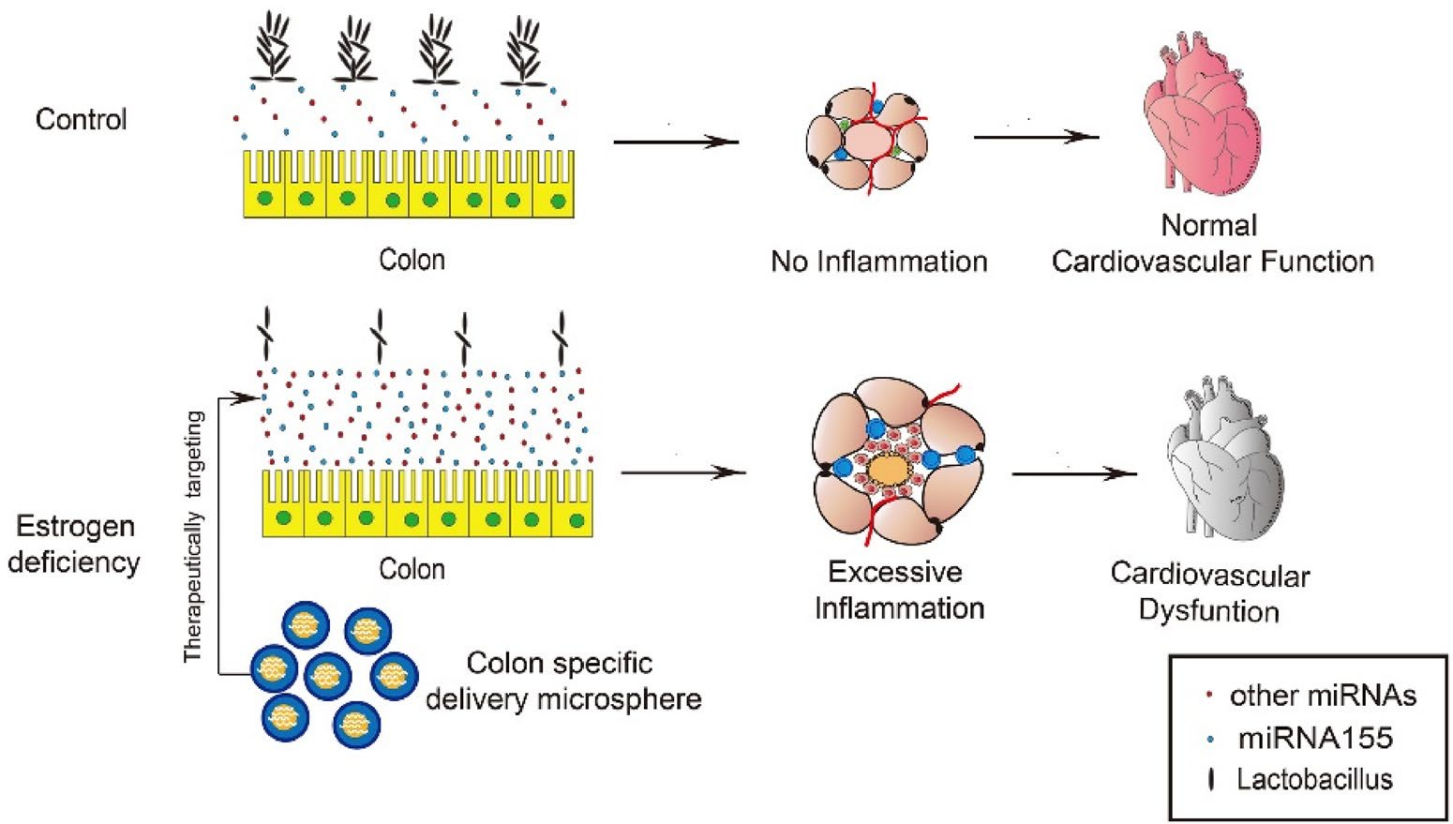
Schematic summary of the study. miR-155 is significantly upregulated in the intestinal epithelium and thus the feces of OVX mice, which in turn remodels the microbiota, especially reducing *Lactobacillus* abundance. Remodeled microbiota results in activated inflammation in both adipose tissue and the heart, contributing to the cardiac dysfunction. Oral delivery of miR-155 antagonism encapsulated in the Eudragit S100 functionalized microspheres restores the beneficial microbiota and thus protects the cardiac function.

Consistent with the restored *Lactobacillus*, the expression of *Tnfα, Mip1,* and *Mcp1* in the adipose tissue and heart decreased significantly after 8 weeks of the anti-miR-155 treatment (Figure 6B, C). Similarly, immune cell infiltration (Figure 6D, E) and ROS generation in the heart was also reduced (Figure S8A, B). Accordingly, the cardiac function was also restored, as shown by the increased EF and E/A values (Figure 6F, G). Moreover, antibiotics treatment nearly blunts the protective effects of the miR-155 antagonism, although antibiotics itself had minor detrimental effects on the inflammation and cardiac function (Figure 6B–G, S7G, H, S8A, B).

## Discussion

CVD is now considered as the main cause of morbidity and mortality in developed countries (Tsao et al., 2022; Timmis et al., 2022). It is well established that the risk for CVD increases in women after menopause (Maas et al., 2021), which might be attributed to hormone change related obesity and MetS (Barros & Gustafsson, 2011). In the present study, we assessed the relationship between intestinal bacteria and cardiovascular function. The major findings of the study were summarized in Figure 7, which include: 1) Decrease of *Lactobacillus* in the gut of OVX mouse model contributes to the increase of visceral fat mass, activated local and systemic inflammation, and thus cardiac dysfunction; 2) estrogen deficiency induced upregulation of miR-155 in the intestinal epithelium and thus the feces is capable to be endocytosed by bacteria in the intestine, which in turn shape the microbiota toward a detrimental signature; 3) Oral delivery of miR-155 antagonists by Eudragit S100 functionalized microspheres restores the protective microbiota and thus cardiac function.

Gut microbiota has been recently linked to CVD (Tang et al., 2019), while the detailed mechanisms regarding how microbiota changes and how changed microbiota alters the cardiovascular system, remain poorly understood. In this study, we found that estrogen deficiency results in altered miRNA profile in the intestine epithelium and thus the feces, which is consistent with previous findings that estrogen/estrogen receptor signal acts as a potent gene expression regulator (Nagarajan et al., 2014). Moreover, our study further revealed that the secreted miRNAs from the intestine not only acts as indicator of the intestine health status, but also acts as an active regulator of the microbiota. Consistent with previous findings (Liu et al., 2016), we found that the altered miRNAs could be uptake by the bacteria, which in turn alters the microbiota diversity. Among the functional miRNAs in the feces, we identified that increase of miR-155 at least partially contributes to the detrimental signature of the gut microbiota in the OVX model. Accumulating studies have suggest that the gut microbiota correlates with the health status (Fan & Pedersen, 2021; de Vos et al., 2022), while the mechanisms are largely unknown. Our study here sets a good example how host health status alters the microbiota. For example, MetS is a prevalent and steady increase risk factor for CVD (Park et al., 2019). Obesity is the most important hallmark of MetS, which is considered to influence and reciprocally be influenced by gut microbiota (Aron-Wisnewsky et al., 2021; Ng et al., 2022). It is thus interesting to analyze the host factors shaping the microbiota in obesity and/or other MetS contexts. As to how increased miR-155 specifically reduced the *Lactobacillus* population, we hypothesize that miR-155 might specifically interact with the gene expression in the *Lactobacillus* via the abundant recognition sites in the genome, and thus interfere with the growth or survival. Future study using mutated miR-155 and RNA-seq analysis of the transcriptomic is would possibly confirm the assumption.

The OVX mice develop metabolic alterations that closely delineate the human MetS, such as dyslipidemia, obesity, and inflammation (Shinlapawittayatorn et al., 2022). Mechanistically, mice with ovariectomy are more prone to accumulate fat due to the lack of hormonal protection. The increase in adipose tissue mass is associated with activated inflammation, which in turn links to higher cardiovascular risk (Oikonomou & Antoniades, 2019). Majority of previous studies have reported that cardiac systolic function is not altered in OVX mice, which is different from our study. However, mechanism studies have suggested that the cardiomyocytes were damaged to some extent under OVX or estrogen deprivation (Ren et al., 2003; Rattanasopa et al., 2015). In fact, there are also some studies revealing that the systolic function is compromised in OVX mice, especially when the mice had additional cardiovascular risks (Jia et al., 2017; Minta et al., 2018). The discrepancy might be due to the differences in echocardiography examination conditions and sensitivity. Besides the variation of culture conditions, such as the growth time and feeding diet, might also accounts. Of note, dead *Lactobacillus* may also show the similar but compromised anti-inflammatory effects as previously reported (Uchinaka et al., 2018).

In this study, we found that miR-155 related reduction of *Lactobacillus* contributes to the increased fat accumulation, systemic inflammation, and thus cardiac dysfunction. However, we could not exclude the involvement of other microbial organisms. Previous studies have revealed that the metabolites from detailed bacteria are the main contributor of inflammation. For example, the choline-derived metabolite TMAO levels are strongly associated with atherosclerosis and cardiovascular risks (Tang et al., 2014; Wang et al., 2015). It is also reported that the gut microbiota is closely related to energy harvest and expenditure (Heiss & Olofsson, 2018; Fluhr et al., 2021). Increase in Firmicutes was significantly correlated to weight gain but not to total caloric intake (Turnbaugh et al., 2006), while *Lactobacillus* is considered to be beneficial for diminishing systemic and local inflammation (Pena & Versalovic, 2003). Our data here raise the possibility that the microbiota might be remodeled toward an obesity resistant signature at the sacrifice of *Lactobacillus* reduction. Our study also raises that *Lactobacillus* would beneficial for OVX related cardiac dysfunction via relieving inflammation. It is also interesting to explore whether *Lactobacillus* is beneficial to other context related MetS and associated cardiovascular dysfunction, such as high fat diet and genetic related obesity. Of note, the phenotype observed in OVX group could be either from reduction of anti-inflammatory microbiota or increase of pro-inflammatory microbiota. Since proteobacteria increased dramatically in the OVX group, it is highly possible that proteobacteria might cause inflammation in OVX mice, which is worth of further study.

In this study, we found that miR-155 increase in OVX intestinal epithelium, which in turn promotes local and systemic inflammation via microbiota dysbiosis. Previously, miR-155 was found to be activated by inflammation in multiple systems (Wang et al., 2017; Jiang et al., 2019). These evidences together suggest a central moderator role of miR-155 in the feedback loop of inflammation-microbiota dysbiosis. It is important to note that besides miR-155, other miRNAs and defensins might be also involved in the estrogen deficiency related microbiota remodeling. It is also important to note that the effects of miR-155 delivery in Sham mice are not as obvious as the effects of anti-miR-155 delivery in OVX mice, which might be explained by potential synergistic role of miR-155 and omental adipose inflammation. Since miR-155 expression increase should be also observed in some other conditions, the observed miR-155-microbiota remodeling should be also related to these conditions with miR-155 highly expressed in the intestine.

Regarding the easy manipulation and targetable traits of miRNAs, we explored the possibility of colon specific delivery of miR-155 antagonist in restoring the microbiota dysbiosis and the associated cardiac dysfunction. The Eudragit S100 coating microsphere is intact in the stomach and intestine and thus protects the miRNA antagonists from degradation in the stomach and small intestine, resulting in specific release of the miRNAs in the colon (Jose et al., 2011). Compared with microbial transplantation, colon specific miRNA delivery-based microbiota restoration has multiple advantages. 1) The intestinal epithelium and the intestinal mucosal barrier that it secretes play a supportive role for the gut flora establishment (Faderl et al., 2015). Bacterium transplantation alone without supportive environment reconstruction might only have transient effects. However, targeting the mechanism how microbiota changes, might reestablish the microbial ecosystem from the preexisting microbiota. 2) Regarding the fecal origin, delivery of microbiota or specific bacterium is psychologically difficult for certain patients. In addition, as a live organism, the bacterium is difficult for manipulation, expansion, and transportation (Browne et al., 2016). In contrast, microsphere or other colon specific delivery strategies based miRNA delivery would overcome all of these disadvantages.

## Conclusions

By using OVX mouse model, we for the first time revealed that altered colon epithelium-derived fecal miRNAs (mainly increased miR-155) were one of the causes of diminished *Lactobacillus* in the gut of the OVX mice. Orally colon specific delivery of miR-155 antagonist at least partially restored the microbiota and exerted a significant cardiac protective role, together with decreased visceral fat deposition, reduced local and systemic inflammation. The current study has established a possible regulatory axis of intestinal miRNAs-microbiota-cardiovascular function in the OVX model. Colon specific delivery of therapeutic miRNAs would possibly restore the microbiota toward protective phenotype in the context of metabolic syndrome.
